# Electrochemical Deposition of Pure-Nickel Microstructures with Controllable Size

**DOI:** 10.3390/mi13050704

**Published:** 2022-04-29

**Authors:** Xiaolei Bi, Lingchao Meng

**Affiliations:** 1 School of Mechanical Engineering, Henan Institute of Technology, Xinxiang 453003, China; 2 State Key Laboratory of High Performance Complex Manufacturing, Central South University, Changsha 410083, China; 3 School of Civil Aviation, Northwestern Polytechnical University, Xi’an 710072, China

**Keywords:** electrochemical deposition, pure nickel, rectangular mandrel, terahertz micro-cavity components, controllable size

## Abstract

Pure nickel microstructures have been widely used in MEMS and have great application potential as a sacrificial mandrel for fabricating terahertz micro-cavity components. The performance of MEMS and terahertz micro-cavity components can be significantly improved through the use of high-quality pure nickel microstructures. Up to now, microfabrication techniques, such as laser micromachining, wire electrical-discharge machining, and cold-spray additive manufacturing, have been used to machine various types of such microstructures. However, huge challenges are involved in using these micromachining techniques to fabricate pure-nickel microstructures with controllable size and good dimensional accuracy, surface roughness, and edge radius. In this paper, taking the example of a pure-nickel rectangular mandrel that corresponds to the size of the end face of a 1.7-THz rectangular waveguide cavity, the machining processes for the electrochemical deposition of pure-nickel microstructures with controllable size, high dimensional accuracy, and good surface roughness and edge radius are discussed systematically. This proposed method can be used to manufacture various types of high-quality pure-nickel microstructures.

## 1. Introduction

Pure nickel is used widely in the preparation of various metal microstructures because of its high ductility, strength, and fatigue and corrosion resistances and superior magnetoelasticity [1,2]. Various types of pure-nickel microstructures have been used successfully in micromachines and microsystems, such as the microscopic coil springs of semiconductor devices [3], the microgear reducer of a microscopic transmission system [4], the microrotor of a microgyroscope [5], and the microcantilevers of hydrogen sensors [6]. Recently, the integral fabrication of a high-working-frequency terahertz rectangular waveguide cavity was reported, and this novel process depends on the manufacture of a pure-nickel sacrificial rectangular mandrel and its selective chemical dissolution [7,8]. The transmission of terahertz signals can be improved significantly through such fabrication of such a cavity, and so a pure-nickel sacrificial rectangular mandrel with controllable size and good surface roughness and fillet radius has great application potential in the manufacturing of terahertz microcavity components.

Various technologies are currently available for machining pure-nickel microstructures. Song et al. studied wire electrical-discharge machining experimentally and manufactured complex pure-nickel parts at microscale and mesoscale using the optimal combination of machining parameters [9]. Hendijanifard et al. studied the Marangoni flow and phase explosion during the laser micromachining of pure nickel and machined microholes in pure-nickel films [10]. However, the influences of heat-affected zones, residual stresses, and melting layers mean that those two methods inevitably have some drawbacks. Cormier et al. fabricated pure-nickel pyramidal fin arrays using cold-spray additive manufacturing, but that approach fell short of achieving high dimensional accuracy and good surface accuracy [11]. Bi et al. obtained a pure-nickel rectangular mandrel with controllable size, high dimensional accuracy, good surface roughness and fillet radius by wire electrochemical micromachining, but the low machining efficiency of that method is not conducive to the mass production of pure-nickel rectangular mandrels [8]. Therefore, it is necessary to explore other types of micromachining technology for pure-nickel microstructures.

Electrochemical deposition (ECD) is a typical additive micromachining technology in which the product is formed layer by layer, and ECD technology based on an aqueous solution generally has the advantages of a wide range of application materials, low operating temperature, coordinated control of microstructure, morphology, and properties, and flexible application form, among others [12,13]. Theoretically, when the metal atoms or grains formed by the reduction reaction are stacked in a controlled manner as designed, metal-based structures and parts of any shape can be fabricated by ECD [14]. With increasing application requirements in the fields of microelectromechanical systems and terahertz devices, ECD has gradually been recognized as a mature micromachining technology to meet these high-precision requirements [15,16].

In this paper, an ECD method is proposed for fabricating pure-nickel microstructures with controllable size, high dimensional accuracy, and good surface roughness and edge radius. Taking the example of fabricating a pure-nickel rectangular mandrel that corresponds to the size of the end face of a 1.7-THz rectangular waveguide cavity, the manufacturing methods are described in detail together with the corresponding experimental investigations.

## 2. Materials and Methods

### 2.1. Materials

In this study, a dry-film photoresist (GPM220; DuPont, Wilmington, DE, USA) (hereinafter referred to simply as the photoresist) with a thickness of 150 μm was selected to prepare the mask with rectangular grooves, and a plate made of 304 stainless steel with a diameter of 100 mm and a thickness of 1 mm was selected as the substrate. A pure nickel plate is used as the anode. The composition of the electrochemical deposition solution of pure nickel is given in Table 1.

### 2.2. Methods

The process for manufacturing the rectangular mandrel is divided into two steps: (i) preparing the mask with rectangular grooves and (ii) the ECD of the rectangular mandrel. The end-face width of the rectangular mandrel is determined by the width of the mask, the length of the rectangular mandrel is determined by the length of the mask, and the end-face thickness of the rectangular mandrel is determined by the time of ECD.

**Figure 1:**
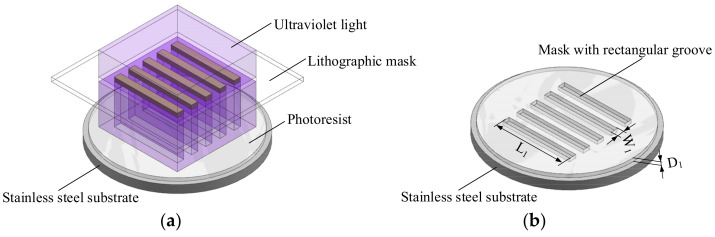
Schematics of key processes during preparation of mask with rectangular grooves: (**a**) exposure; (**b**) development.

The mask is manufactured by lithography, including substrate treatment and photoresist coating, baking before exposure, exposure, baking after exposure, and development. The length and width of the mask are controlled through the photomask, and the end-face height of the mask is guaranteed by the thickness of the photoresist; the latter selected for use is greater than the end-face thickness of the rectangular mandrel prepared by ECD. The key processes of exposure and development during the preparation of the mask are shown in Figure 1. The thickness of the mask is *D*_1_, its length is *L*_1_, and its width is *W*_1_. The dimensional accuracy and side surface roughness of the mask are guaranteed mainly by the exposure and development parameters, and its bottom surface roughness is guaranteed by the surface roughness of the substrate.

**Figure 2:**
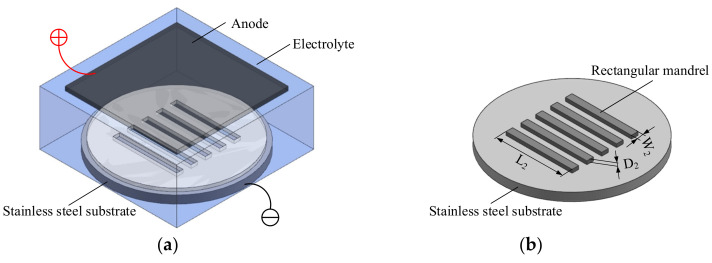
Schematics of manufacturing rectangular mandrel by ECD: (**a**) process; (**b**) result.

The ECD of the rectangular mandrel is shown in Figure 2. The end-face thickness of the finally prepared rectangular mandrel is *D*_2_, its length is *L*_2_, and its end-face width is *W*_2_. The dimensional accuracy, side roughness, and edge radius of the rectangular mandrel are guaranteed mainly by the accuracy of the mask and the ECD parameters, and the bottom surface roughness of the rectangular mandrel is determined by the surface roughness of the substrate. The length *L*_2_ and end-face width *W*_2_ of the finally prepared rectangular mandrel are consistent with the length *L*_1_ and width *W*_1_ of the mask, and the thickness *D*_2_ of the end face of the rectangular mandrel is less than the thickness *D*_1_ of the mask.

## 3. Results

### 3.1. Preparation of Mask with Rectangular Grooves

#### 3.1.1. Substrate Treatment

The surface quality of the lower surface of the rectangular mandrel is guaranteed by the surface quality of the substrate. Therefore, if surface treatment of the substrate is not carried out, then various defects on the substrate surface will be copied on the lower surface of the rectangular mandrel. Moreover, impurities, such as oil stains and particles on the substrate surface, will make the photoresist unable to fit closely with the substrate, resulting in the falling off of the mask in the subsequent ECD process and the deposition of metal on the lower surface of the rectangular mandrel beyond the groove edge area after ECD. Therefore, surface treatment of the substrate is very important.

**Figure 3:**
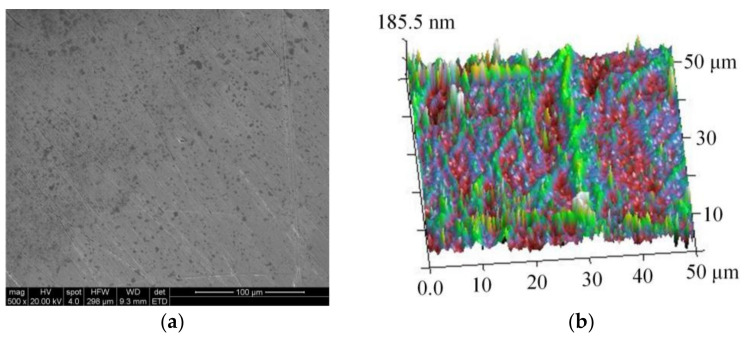
Observation examples of substrate surface morphology: (**a**) scanning electron microscopy (SEM); (**b**) atomic force microscopy (AFM).

First, the substrate surface is polished precisely to remove the macroscopic and microscopic defects on its surface. Then, the substrate is put into acetone solution for ultrasonic cleaning to remove the surface oil stains and adsorbed impurities, and then the substrate is immersed in 0.1-mol/L dilute hydrochloric acid solution for 30 s. Finally, the substrate is cleaned ultrasonically with deionized water and then dried for use. To verify the effect of substrate treatment, the substrate surface roughness must be measured, during which two different positions on three substrates are selected randomly. The specific measurement results are given in Table 2, and scanning electron microscopy (SEM) and atomic force microscopy (AFM) observations of the substrate surface morphology are shown in Figure 3.

#### 3.1.2. Photoresist Coating and Baking before Exposure

The aim in photoresist coating is to apply it tightly and evenly on the substrate surface, so it is necessary to perform the process via a laminator. To achieve the film-coating effects of a flat, uniform, and bubble-free surface, it is necessary to apply the photoresist with a certain pressure and temperature, and in the present study the laminating pressure was 1.5 kg/cm^2^.

**Figure 4:**
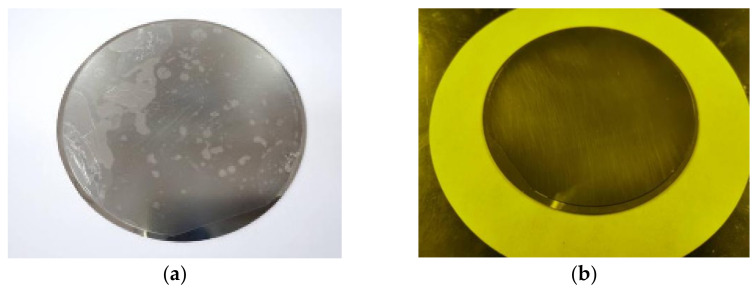
Results of photoresist coating at different temperatures: (**a**) laboratory temperature; (**b**) 60 °C.

A dry-film photoresist is thin and brittle and so is easily damaged when a certain pressure is applied directly by the laminator, as shown in Figure 4a. Therefore, it is necessary to set the laminating roller to a certain temperature first so that it preheats the photoresist when close to it. After the photoresist becomes soft, one tears off the polyethylene protective film on one side and quickly sticks it on the substrate. Controlling a certain temperature during coating softens the photoresist by heating, thereby making it fit the substrate more easily. Therefore, the photoresist preheating temperature, i.e., the temperature of the laminating roller, has a great impact on the coating quality. Via previous experimental exploration, 60 °C was selected for the preheating and coating of the photoresist, and the workpiece after coating is shown in Figure 4b.

After the photoresist coating is completed, baking before exposure must be done. The purpose of this is to increase the fluidity of the photosensitive adhesive in the photoresist so that the photoresist and the substrate fit more closely and the photoresist surface is flatter and more uniform, with the overall aim being to obtain better exposure results. Before the baking before exposure, the polyethylene protective film on the other side of the photoresist surface must be torn off. To obtain better baking, it is necessary to (i) set the baking temperature and time so that the temperature rises slowly and (ii) allow the temperature to drop slowly for cooling after baking. Via previous experimental exploration, the chosen temperature and time settings for baking before exposure were as given in Table 3.

#### 3.1.3. Exposure and Baking after Exposure

Currently, the main photoresist-exposure methods are proximity, contact, and projection, and contact exposure was used in the present study. During exposure, the photoresist surface is in direct contact with the photomask, and the designed pattern on the photomask is copied onto the photoresist in the ratio of 1:1. Because the light-source radiation intensity of the exposure machine used in the present study was stable, the exposure accuracy was determined mainly by the exposure time. If the exposure time is too short, then the photoresist layer cannot be fully sensitized, and the crosslinking reaction is insufficient. During subsequent development, the photoresist at the edge of the exposure area will also dissolve in the developer, resulting in an uneven or large final mask relative to its design size. If the exposure time is too long, then the photoresist at the mask will produce a cross-linking reaction due to light scattering or diffraction reaction, resulting in excessive exposure. During subsequent development, the photoresist near the edge of the mask cannot dissolve fully in the developer, resulting in the actual mask being small or there being more photoresist residues at the groove edges. Therefore, choosing a reasonable exposure time is the key to high-precision copying of the mask design pattern.

**Figure 5:**
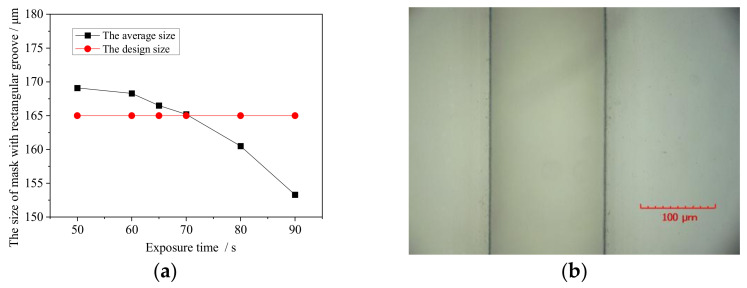
Exposure results: (**a**) average mask size relative to design size with different exposure times; (**b**) local morphology of mask with exposure time of 70 s.

In the present study, the end-face size of the 1.7-THz waveguide cavity was 83 μm × 165 μm, so the design width of the mask was 165 μm. Figure 5a shows how the average size of the mask relative to the design size varied with the exposure time. With increasing exposure time, the actual size of the mask decreases gradually. When the exposure time is 70 s, the consistency of the obtained mask is good, and its average size is close to the design size. Figure 5b shows the experimental results for an exposure time of 70 s, which was selected as the best exposure time in the present study.

After exposure, baking is required, the purpose being to accelerate the cross-linking reaction of the photoresist in the exposure area and make the exposure area more stable. However, excessive post-baking will increase the stress in the adhesive film and even crack and deform it, while baking at too low a temperature and for insufficient time will lead to insufficient cross-linking reaction of the photoresist and poorer quality of the mask after subsequent development. The chosen temperature and time settings for baking after exposure were as given in Table 4.

#### 3.1.4. Development

Development is a key step in the preparation of the mask, the purpose being to dissolve the unexposed areas in the photoresist and retain the exposed ones to obtain the rectangular grooves of the template. In this study, during development, the exposed workpiece was put into the developer for static development, and the developer was shaken and stirred every 30 s. Because the unexposed photoresist reacts with isopropyl alcohol solution and produces white precipitation, it can be judged whether the development is complete by putting the developed workpiece into isopropyl alcohol solution.

**Figure 6:**
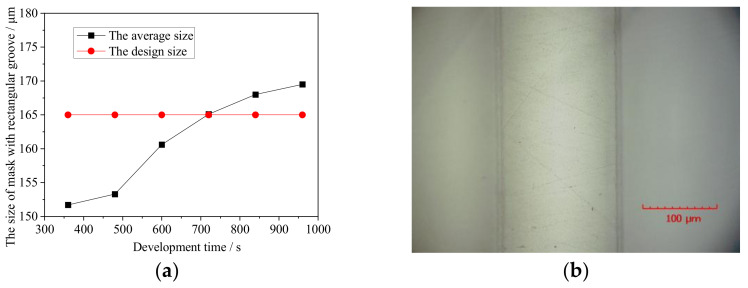
Development results: (**a**) average mask size relative to design size with different development times; (**b**) local morphology of mask with development time of 720 s.

With insufficient development time, the photoresist in the unexposed areas cannot dissolve fully, leading to poor edge uniformity of the mask due to residual photoresist. With excessive development time, although the photoresist in the unexposed areas dissolves fully, the developer penetrates into the exposed areas at the mask edges, causing them to swell and lowering the uniformity of the groove edge size of the mask and causing the edges to lose their luster. Therefore, reasonable control of the development time is also needed for mask accuracy. After exposure for 70 s, different development times were selected, and Figure 6a shows how the average size of the mask relative to the design size after development varied with the development time. It is found that a development time of 720 s gives clean photoresist development and good mask size and accuracy, the mask being close to its design size. Figure 6b shows the experimental results for a development time of 720 s, which was chosen as the development time in the present study.

#### 3.1.5. Results of Preparing Masks with Rectangular Grooves

**Figure 7:**
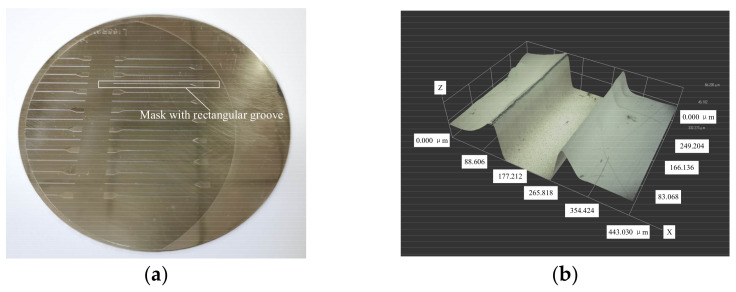
Results of preparing mask with rectangular grooves: (**a**) two-dimensional overall view; (**b**) local three-dimensional topography.

**Figure 8:**
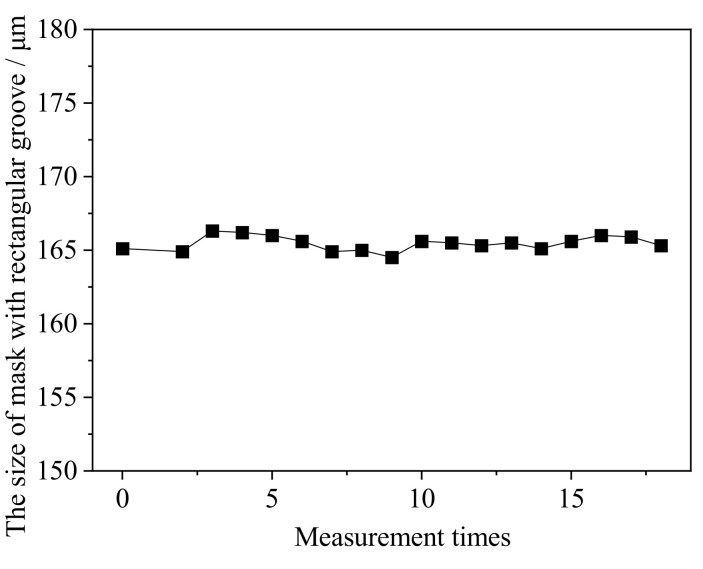
Variation of measurements of mask with rectangular grooves.

Using the optimized combination of process parameters, masks with a design width of 165 μm were prepared, and Figure 7 shows an example of their morphology. Six different workpieces were randomly selected for measurement, and three masks in each workpiece were randomly selected for size measurement. Figure 8 shows how the measurements varied, and accordingly the average mask width was 164.5 μm.

### 3.2. Electrochemical Deposition of Rectangular Mandrel

**Figure 9:**
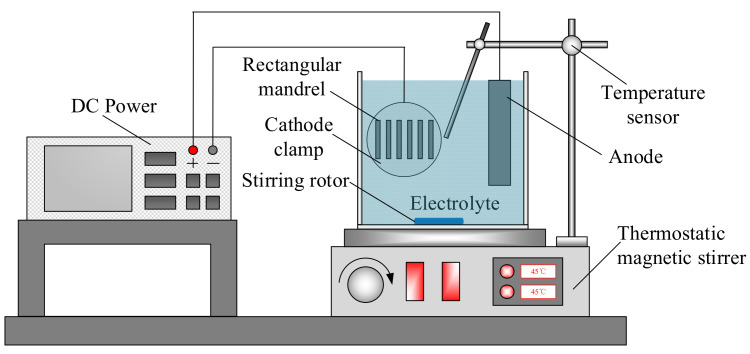
Schematic of experimental platform for ECD.

Figure 9 shows the experimental platform used for the ECD of the rectangular mandrel in the mask. A DC power supply with a resolution of 0.01 mA was used, and an air-bearing spindle was used to rotate the cathode. Agitation and temperature control of the solution were realized with a temperature-controlled magnetic stirrer. During the experiment, the stirrer controlled the magnetic rotor to maintain constant rotation to stir the ECD solution and improve its renewal flow around the cathode. The experimental conditions are given in Table 5.

**Figure 10:**
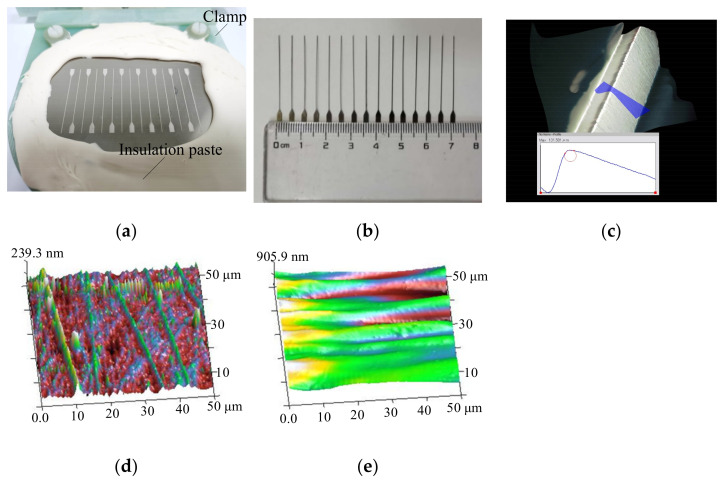
ECD results of pure-nickel rectangular mandrel: (**a**) overall morphology of workpiece after ECD; (**b**) pure-nickel rectangular mandrel; (**c**) edge morphology; (**d**) AFM morphology of bottom surface; (**e**) AFM morphology of side surface.

According to the end-face size of the rectangular mandrel corresponding to the 1.7-THz rectangular waveguide cavity, the ECD time in the present study was set to 8 h. Figure 10a shows the workpiece after ECD, and Figure 10b shows the obtained pure-nickel rectangular mandrels. The method for measuring the rectangular-mandrel width was to randomly select a rectangular mandrel and randomly select six positions along its length for measurement. The method for measuring the rectangular-mandrel fillet radius and surface roughness was the same as that of the width. Figure 10c–e show observations of the rectangular-mandrel edge, bottom, and side morphologies, respectively, and Table 6 gives the results of measuring the technical indices of the width, edge radius, bottom surface roughness, and side surface roughness of the pure-nickel rectangular mandrel.

## 4. Conclusions

An ECD machining process for pure-nickel microstructures with controllable size, high dimensional accuracy, and good surface roughness and edge radius was investigated and discussed systematically, the following conclusions were obtained.

In the process of preparing the mask with rectangular grooves, an exposure time of 70 s and a development time of 720 s were considered to be optimal choices in the present study.

During the electrochemical deposition, the temperature of the solution was kept at 45 °C to give the best reaction rate with a current density of 1.0 A/dm^2^. The pH of the electrochemical deposition solution was controlled to be in the range of 3.5–4.5.

The width of the final prepared rectangular mandrel is consistent with the mask. The measurement results show that the bottom surface roughness is less than 0.1 μm, the side roughness is less than 0.2 μm, and the edge radius is less than 9.2 μm.

Because the specific size is controllable and the dimensional accuracy, surface roughness, and edge radius are good, the proposed method can be used to manufacture various types of high-quality pure-nickel microstructures.

## Figures and Tables

**Table 1:** Composition of the electrochemical deposition solution of pure nickel.

Component	Concentration (g/L)
Nickel sulfamate	450
Nickel chloride	15
Boric acid	30
Sodium dodecyl sulfate	0.1

**Table 2:** Measurement results of surface roughness of substrate.

Measuring Position	1	2	3	4	5	6
*R*_a_ [μm]	0.0532	0.0473	0.0516	0.0462	0.0573	0.0495

**Table 3:** Settings for baking before exposure.

Step	Temperature [°C]	Time [s]
1	65.0	600
2	95.0	600
3	95.0	1200
4	30.0	3600

**Table 4:** Settings for baking after exposure.

Step	Temperature [°C]	Time [s]
1	65.0	600
2	95.0	600
3	95.0	1200
4	30.0	3600

**Table 5:** Experimental conditions for ECD.

Parameter	Value
Solution temperature	45 °C
pH	3.5–4.5
Current density	1 A/dm^2^

**Table 6:** Measured values of technical indices.

Parameter	Values					
Position	1	2	3	4	5	6
Width [μm]	164.3	163.9	163.2	164.1	164.6	163.7
Bottom surface roughness [μm]	0.0617	0.0538	0.0522	0.0596	0.0536	0.0639
Side surface roughness [μm]	0.126	0.157	0.118	0.180	0.176	0.137
Edge radius [μm]	8.327	8.911	8.723	8.632	9.176	8.251
